# N6-Isopentenyladenosine Hinders the Vasculogenic Mimicry in Human Glioblastoma Cells through Src-120 Catenin Pathway Modulation and RhoA Activity Inhibition

**DOI:** 10.3390/ijms221910530

**Published:** 2021-09-29

**Authors:** Cristina Pagano, Giovanna Navarra, Olga Pastorino, Giorgio Avilia, Laura Coppola, Rosa Della Monica, Lorenzo Chiariotti, Tullio Florio, Alessandro Corsaro, Giovanni Torelli, Pasquale Caiazzo, Patrizia Gazzerro, Maurizio Bifulco, Chiara Laezza

**Affiliations:** 1 Department of Molecular Medicine and Medical Biotechnology, University of Naples “Federico II”, 80145 Naples, Italy; pagano.cris@gmail.com (C.P.); vanna.navarra@libero.it (G.N.); olga.pastorino@gmail.com (O.P.); giorgioavilia8@gmail.com (G.A.); coppola.laura6@gmail.com (L.C.); dellamonica@ceinge.unina.it (R.D.M.); chiariot@unina.it (L.C.); 2 CEINGE—Biotecnologie Avanzate, Via Gaetano Salvatore 486, 80145 Naples, Italy; 3 Department of Internal Medicine, University of Genova, Viale Benedetto XV 2, 16136 Genova, Italy; tullio.florio@unige.it (T.F.); alessandro.corsaro@unige.it (A.C.); 4 IRCCS Ospedale Policlinico San Martino, Largo R. Benzi 10, 16132 Genova, Italy; 5 Neurosurgery Unit A.O. San Giovanni di Dio e Ruggi d’Aragona—Salerno’s School of Medicine Largo Città di Ippocrate, 84131 Salerno, Italy; giovanni.torelli@sangiovannieruggi.it; 6 Neurosurgery, Unit A.O. “A.Cardarelli”, 80145 Naples, Italy; pasquale.caiazzo@aocardarelli.it; 7 Department of Pharmacy, University of Salerno, Fisciano, 84084 Salerno, Italy; pgazzerro@unisa.it; 8 Institute of Endocrinology and Experimental Oncology (IEOS), National Research Council (CNR), 80145 Naples, Italy

**Keywords:** N6-isopentenyladenosine, vasculogenic mimicry, c-Src kinase, E-cadherin, RhoA

## Abstract

Background: Vasculogenic mimicry (VM) is a functional microcirculation pattern formed by aggressive tumor cells. Thus far, no effective drugs have been developed to target VM. Glioblastoma (GBM) is the most malignant form of brain cancer and is a highly vascularized tumor. Vasculogenic mimicry represents a means whereby GBM can escape anti-angiogenic therapies. Methods: Here, using an in vitro tube formation assay on Matrigel, we evaluated the ability of N6-isopentenyladenosine (iPA) to interfere with vasculogenic mimicry (VM). RhoA activity was assessed using a pull-down assay, while the modulation of the adherens junctions proteins was analyzed by Western blot analysis. Results: We found that iPA at sublethal doses inhibited the formation of capillary-like structures suppressing cell migration and invasion of U87MG, U343MG, and U251MG cells, of patient-derived human GBM cells and GBM stem cells. iPA reduces the vascular endothelial cadherin (VE-cadherin) expression levels in a dose-dependent manner, impairs the vasculogenic mimicry network by modulation of the Src/p120-catenin pathway and inhibition of RhoA-GTPase activity. Conclusions: Taken together, our results revealed iPA as a promising novel anti-VM drug in GBM clinical therapeutics.

## 1. Introduction

Glioblastoma is the most malignant and aggressive primary brain cancer in adults having a poor prognosis despite surgery and conventional radio-chemotherapy. GBM is an extensive vascularized tumor, a process required to sustain growth and invasion of the cancer; moreover, anti-angiogenic therapy has limited efficacy due to acquired anti-angiogenic resistance [1,2]. Recent research has described that in brain tumors there is an alternative vascularization process, referred to as vasculogenic mimicry (VM), formed by tumor cells by acquiring plasticity to mimic endothelial function associated with tumor highly invasive. VM channels create a network of vessels connecting with those of the endothelium allowing invasion and metastasis [3]. The molecular mechanism of VM channel formation is poorly studied in GBM. Numerous studies have observed that anti-angiogenic therapy in GBM may also be incapable of inhibiting VM suggesting a poor response at this therapy [4,5,6]. Important roles in angiogenesis, embryogenesis, and morphogenesis are played by cell adhesion molecules of the cadherin superfamily, in particular by E- and VE-cadherin. Cadherins with their associated proteins, catenins, represent the cornerstone of adherens type junctions in the endothelium as well as in the epithelium. The interactions with members of the armadillo family as β-catenin and p120-catenin (p120) regulate the association of cadherins with the actin cytoskeleton [7,8]. Furthermore, key roles of the catenins are hypothesized to control E-cadherin clustering, recycling, Rho-GTPase modulation, cytoskeleton remodeling, as well as transcriptional control. In particular, Rho GTPases are necessary to regulate actin stress fiber formation mediated by ROCK kinase (Rho-associated protein kinase) [9]. A recent study described that the RhoA/ROCK pathway was involved in the VM process in a hepatocellular carcinoma (HCC) cell line [10]. N6-isopentenyladenosine (iPA), an adenosine modified with an isopentenyl chain, a product of the mevalonate pathway, exerts an antiproliferative effect against various tumors [11,12,13] including glioblastoma in vitro and in vivo models [14,15]. We also observed that it inhibits all the steps of the angiogenic process in vitro and in mouse models. Indeed, treatment with iPA strongly blocked endothelial cell proliferation in a dose- and time-dependent manner [16]. Here, we demonstrated that iPA inhibits VM affecting the cytoskeletal structure of the GBM cells inhibiting their migration, and blocking the formation of tube-like structures on ECM (extracellular matrix) in vitro, as a model of vasculogenic mimicry. We examined the mechanism involved, relating the inhibition of the c-Src-120catenin pathway and inhibition of RhoA-GTPase activity.

## 2. Results

### 2.1. N6-Isopentenyladenosine Inhibits the Tube Formation in GBM Cells

**Figure 1:**
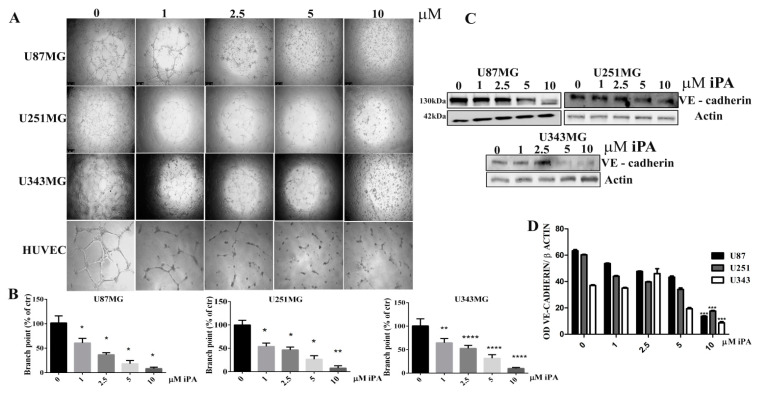
N6-isopentenyladenosine inhibits the tube formation in GBM stabilized cell lines. (**A**) Representative images of vascular tubes formed by cells in control group (DMSO) and in treated group at 18 h. iPA is used at several concentrations. Network of tubes, in each well, were analyzed directly under an inverted microscope with 4× phase contrast and imaged. Scale bars: 250 µm. (**B**) Quantification of vascular branching. Branch point (sites of intersection of at least three tubes) number in each well was counted. (**C**) Immunoblotting showing the effect of iPA on VE-cadherin in GBM stabilized cell lines. (**D**) Densitometric analysis of each Western Blot panel. Results are expressed as the mean ± SD of four independent experiments conducted in triplicate. (ANOVA, * *p* < 0.05, ** *p* < 0.01, *** *p* < 0.001, **** *p* < 0.0001).

Glioblastoma cells are able to form vessel-like networks, characteristic VM structures in tumor tissues contributing functionally to tumor progression [17,18]. When U87MG, U251MG, and U343MG human glioblastoma cell lines are cultivated in 3D-Matrigel^®^ (ECM), under serum-free conditions, they showed considerable tube-forming ability similar to those formed by HUVECs (Human umbilical vein endothelial cells) (Figure 1A). A hallmark of vasculogenic mimicry is VE-cadherin expression while they do not express the endothelial marker CD31 (data not shown) [17,19]. To assess the iPA effect on VM formation, we treated the GBM cells on the Matrigel-coated wells with iPA at concentrations ranging from 1 to 10 µM and then carried out a three-dimensional (3D) culture VM tube formation assay for 18 h. As shown in Figure 1A, the GBM cell lines treated with the vehicle alone formed complete tubular channels within 15 h, in contrast, the tube formation was inhibited by iPA treatment at the same time as well as for HUVEC cells, in vitro model of angiogenesis. VM tube formation of GBM cells was dramatically inhibited by 85% for U87MG, 92% for U251MG, and 89% for U343MG at 10 µM of iPA after 18 h. The total tube area of each group was determined using ImageJ (Figure 1B). To confirm that iPA’s ability to hinder tube formation is not due to cell proliferation inhibition of GMB cells, we performed an MTT assay with various concentrations of iPA (1–10 µM) after 18 h of treatment. We did not observed significant changes in the cell proliferation rate of GBM cells and HUVEC cells treated with iPA (1–10 µM) for 18 h in comparison to the untreated group (Figure S1). To examine the iPA effect on VE-cadherin expression involved in VM formation, we analyzed the protein levels by Western blot in GBM cell lines treated with iPA for 18 h (Figure 1C,D).

**Figure 2:**
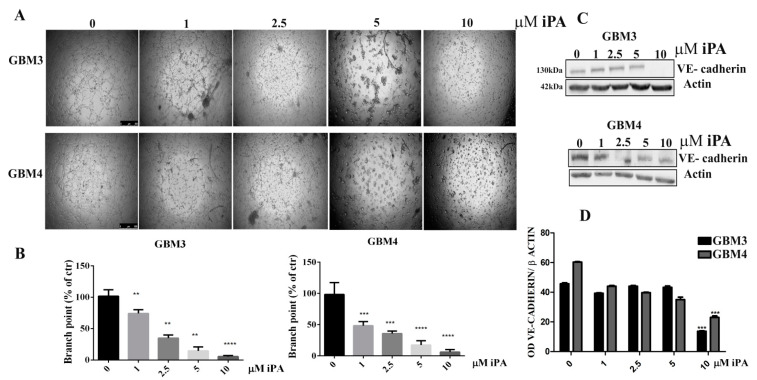
N6-isopentenyladenosine inhibits the tube formation in GBM primary cell lines. (**A**) Representative images of vascular tubes formed by primary cells in control group (DMSO) and in treated group at 18 h. iPA is used at several concentration. Network of tubes, in each well, were analyzed directly under an inverted microscope with 4× phase contrast and imaged. Scale bars: 250 µm. (**B**) Quantification of vascular branching. Branch point (sites of intersection of at least three tubes): the number in each well was counted. (**C**) Immunoblotting showing the effect of iPA on VE-cadherin in GBM primary cell lines. (**D**) Densitometric analysis of each Western Blot panel. Results were expressed as the mean ± SD of four independent experiments conducted in triplicate. (ANOVA, ** *p* < 0.01, *** *p* < 0.001, **** *p* < 0.0001).

VE-cadherin expression was strikingly downregulated by treatment of iPA mostly at 5 and 10 µM in comparison with cells treated with vehicle alone, suggesting that the downregulation of VE-cadherin is associated with iPA-inhibited VM formation in GBM cells. Afterwards, we evaluated the iPA effects on VM of cells isolated from fresh primary tumor resection of glioma patients. The primary GBM cells (GBM3 and GBM4) were characterized for the mutational status of IDH1/IDH2, methylation of MGMT and p53, and EGFR amplification (Table 1) as described in the Materials and Methods. The GBM primary cells in the control group formed vessel-like networks in the Matrigel within 18 h, (Figure 2A). VM tube formation of GBM primary cells was dramatically inhibited by 85% for GBM3 and 92% for GBM4 at 10 µM of iPA at after 18 h of treatment (Figure 2B). Moreover, we observed that iPA do not inhibit the cell viability of the primary cells at 18 h (Figure S1). We analyzed the VE-cadherin protein levels by Western blot in primary cells treated for 18 h with iPA at several concentrations (Figure 2C,D). VE-cadherin protein levels were reduced by treatment with iPA at 10 µM for GBM 3 and at 2.5 to 10 µM for GBM4 in comparison with cells treated with vehicle alone. The GBM cells are negative to endothelial marker CD31 [17] (data not shown). Recently, glioma stem cells (GBM CSC) have been shown to contribute to VM formation as well as angiogenesis. Then, we assessed the iPA effect on VM in CSCs (GBM1 and GBM2), isolated from tumor biopsies of patients affected by GBM [17]. iPA at 10 µM reduced cell ability to form vessel-like networks in the Matrigel after 24 h of treatment (Figure S2A,B). Furthermore, we observed any significant change in cell viability at 10 µM for GBM1 and GBM2 after 24 h of treatment (Figure S2C,D).

### 2.2. Inhibitory Effect of iPA on Cell Motility In Vitro

**Figure 3:**
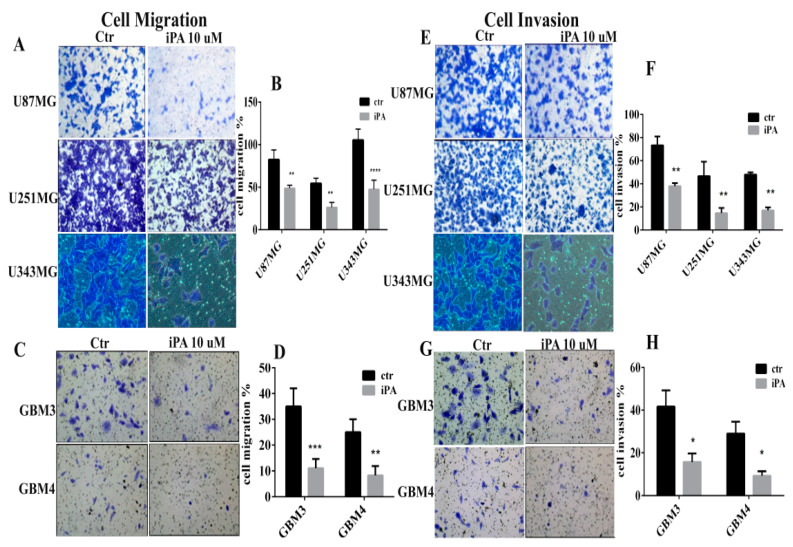
Inhibitory effect of iPA on cell motility in vitro. Transwell assays were used to measure the effect of iPA on cell migration and invasion of GBM cells. (**A**) Representative images showing the reduction of cell migration of cell lines U87MG, U251MG, and U343MG treated with iPA at 18 h, stained with crystal violet. (**B**) Diagram showing the quantitative analysis of U87MG, U251MG, and U343MG cells migration at 18 h. (**C**) Representative images showing the reduction of cell migration GBM primary cell line (GBM3-4) treated with iPA at 18 h, stained with crystal violet. (**D**) Diagram showing the quantitative analysis of GBM primary cell line (GBM3-4) migration at 18 h. (**E**) Representative images showing the reduction of cell invasion of cell lines U87MG, U251MG, and U343MG treated with iPA at 18 h, stained with crystal violet. (**F**) Diagram showing the quantitative analysis of U87MG, U251MG, and U343MG cells invasion at 18 h. (**G**) Representative images showing the reduction in cell invasion of GBM primary cell line (GBM3-4) treated with iPA at 18 h, stained with crystal violet. (**H**) Diagram showing the quantitative analysis of invasion of GBM primary cell line (GBM3-4) at 18 h. The images are shown at 10× magnification. Results are expressed as the mean ± SD of four independent experiments conducted in triplicate. (ANOVA, * *p* < 0.05, ** *p* < 0.01, *** *p* < 0.001, **** *p* < 0.0001).

VM is associated with cell migration and invasion [20]; thus, we evaluated the effects of iPA on migratory and invasion properties of GBM cells using transwell assays. As reported in Figure 3A, iPA treatment at 10µM inhibited cell migration compared to the control at 18 h. The migration was quantified and the data are shown in Figure 3B. Moreover, we tested the capability of iPA to hinder GBM cells to invade ECM. In Boyden chamber invasion assays, at 18 h, iPA reduced cell invasion compared to the control group treated with vehicle alone (Figure 3E). The invasion was quantified and the data are shown in Figure 3F. We observed the same results with GBM primary cells for migration (Figure 3C,D) and invasion (Figure 3G,H).

### 2.3. Effect of iPA on Src-120 Catenin Pathway

**Figure 4:**
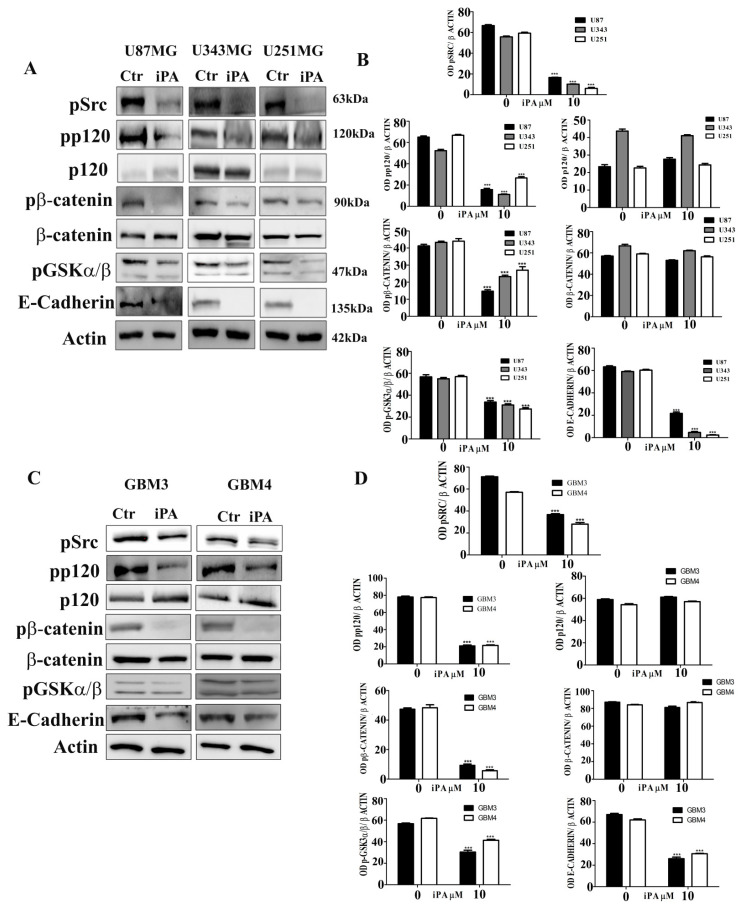
Western Blot analysis was used to assess iPA effect on levels of AJ proteins at 18 h (**A**) iPA reduced pSrc, pp120 catenin, pβ-catenin, pGSK3α/β and E-cadherin protein in U87MG, U343MG, and U251MG stabilized cell lines. (**B**) Densitometric analysis of each Western Blot. (**C**) iPA effect on AJ protein levels at 18 h on GBM primary cell lines (GBM3 and GBM4), iPA reduced pSrc, pp120 catenin, p β-catenin, pGSK3α/β, and E-cadherin protein. (**D**) Densitometric analysis of each Western Blot. Results are expressed as the mean ± SD of four independent experiments conducted in triplicate. (ANOVA, *** *p* < 0.001).

VE-cadherin as well as E-cadherin promote homotypic cell–cell interactions, essential for stabilization of the adherens junctions (AJs) of endothelial or epithelial cells [21]. AJs consist of cadherin adhesion receptors, providing homophilic ligation with cadherins on adjacent cells, and members of the catenin protein family, including p120, β- and α-catenin. The linkage of α-catenins with the actin cytoskeleton constitutes the organization of AJs in different cell types [22]. To assess the iPA effect on the main components of AJs, we evaluated the protein levels of E-cadherin and the phosphorylation status of the p120-catenin and β-catenin by Western blot analysis. We observed that iPA treatment at 10 µM for 18 h reduced the protein levels of E-cadherin, the phosphorylation of the T219/T216 residues phosphorylation of pGSK3α/β which in turn regulates the phosphorylation of the β-catenin. Indeed, IPA treatment inhibited β-catenin at S37/T41 residues in GBM cell lines and primary cells as compared to cells treated with vehicle alone. (Figure 4A–D).

Moreover, we observed that iPA treatment at 10 µM inhibited the phosphorylation of Y228 residues of p120-catenin. Since it has been described that p120-catenin is enriched in Src-family kinase sites within its amino-terminal region, including Y228 [23], we examined whether iPA is able to reduce the c-Src kinase activity through phosphorylation inhibition. We observed that GBM cells treated with iPA reduced the phosphorylation status of Y416 residue of the Src-kinase by specific antibody in comparison to untreated cells suggesting inhibition of c-Src activity (Figure 4A–D). To assess this hypothesis, we evaluated the phosphorylation status of p120 catenin in GBM cell lines and primary cells treated for 18 h with an established Src inhibitor, Dasatinib at 1 µM [24]. We observed that the phosphorylation of Y228 residues of p120 catenin and of the Y416 residue of Src was reduced in the GBM cells treated with Dasatinib compared to untreated GBM cells (Figure S3).

### 2.4. Effect of iPA on RhoA GTPase Activity

**Figure 5:**
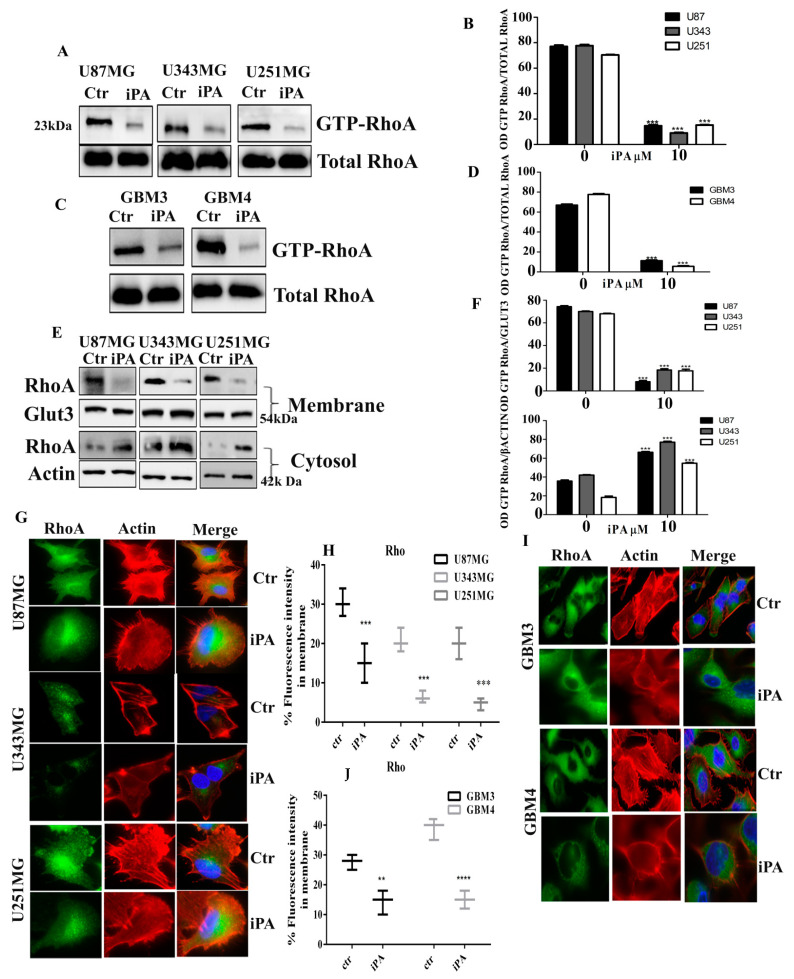
Effect of iPA on RhoA GTPase activity. Western Blot analysis showing the reduction in GTP-RhoA levels in iPA treated (**A**) U87MG, U251, U343 cells and (**C**), GMB primary cells line (GBM 3; GMB 4) at 18 h. (**E**) Western Blot analysis showing RhoA protein levels in membrane/cytosol fractions obtained from iPA treated and untreated GBM stabilized cell lines at 18 h. (**B**,**D**,**F**) Densitometric analysis of each Western Blot panel. Immunofluorescence staining of RhoA (green signal) and Actin (red signal, Phalloidin) in iPA treated and untreated. (**G**) GBM stabilized and (**I**) primary cell lines at 18 h. The Merge panel shows the overlapping of the two signals (green signal + red signal): in iPA treated cells, RhoA is reduced from the membrane. (**H**,**J**) The rate of F-actin was quantified and data are plotted as boxplots. Results are expressed as the mean ± SD of four independent experiments conducted in triplicate. (ANOVA, ** *p* < 0.01, *** *p* < 0.001, **** *p* < 0.0001).

p120-catenin also binds intracellular proteins, such as the small GTPase RhoA upon the phosphorylation of Tyr217 and Tyr228 by Src [22], besides, Rho GTPases are the main regulators of the assembly, dynamics, reorganization, and contractility of the actin cytoskeleton [25]. To assess the iPA effect on the RhoA activity, we analyzed the level of the active GTP-bound form of RhoA by a pull-down assay with the Rho-binding fragment of rhotekin in GBM cells treated with iPA at 10 µM. The cells were developed in serum-free medium for 18 h, and after treatment, we collected the GBM cell lines, GBM3 and GBM4, and performed the assay. As shown in Figure 5 a large proportion of the RhoA protein detected in GBM cells lines (Figure 5A,B) and GBM primary cells (Figure 5C,D) is present in an active form. Upon the iPA treatment for 18 h, the level of GTP-RhoA was decreased (Figure 5A–D). The reduction was statistically significant at 10 µM of iPA compared with the control group. Furthermore, we investigated the effect of Dasatinib on RhoA activation by pull-down assay of the GBM cells untreated or treated for 18 h with 1 µM of the Src kinase inhibitor. We observed that Dasatinib treatment for 18 h induced RhoA activation compared to untreated GBM cells (Figure S4A,B). To determine whether iPA or Dasatinib treatment impaired the p120/RhoA interaction, the ability of p120-catenin to coimmunoprecipitate with RhoA was tested with inhibitors. Lysates of GBM cells were immunoprecipitated by p120 antibodies and the precipitates were immunoblotted with RhoA and phospho-p120 catenin antibodies. Figure S4 shows that RhoA can coimmunoprecipitate with pp120 catenin in untreated GBM cells but not in cells treated for 18 h with 10 µM of iPA (Figure S4C,D) or 1 µM Dasatinib (Figure S4E,F), suggesting that the phosphorylation of Y228-p120 catenin is important for the interaction with RhoA. Moreover, because the RhoA protein must be targeted to the plasma membrane for activation and full function, we examined the translocation of this protein from the cytosol to the cell membrane in GBM cells lines in the presence of iPA at 10 µM, after separation of particulate and soluble fractions. In untreated cells, most of the RhoA protein was present on the cell membrane (Figure 5E,F). Treatment with iPA clearly decreased the amount of RhoA in the membrane fraction and accumulated in the cytosolic fraction for 18 h (Figure 5E,F). As RhoA is involved in the regulation and assembly of contractile actin filaments (stress fibers), we studied the effect of iPA on the actin cytoskeleton by immunofluorescence Thunder microscopy using phalloidin TRITC-conjugated (Tetramethylrhodamine). As shown in Figure 5G,I, various stress fibers were detected in untreated cells. The treatment with iPA at 10 µM for 18 h caused a significant decrease in F-actin-containing stress fibers; many cells displayed a dense meshwork of unpolarized actin filaments around the cell periphery. We also observed the intracellular distribution of RhoA with a specific monoclonal antibody and a secondary antibody FITC-conjugated (Fluorescein isothiocyanate). Interestingly, RhoA was present at the membrane periphery in untreated cells, while in the cells treated with iPA, the RhoA patches to the cell membrane were reduced (Figure 5H–J). RhoA remained largely diffused in the cytoplasm mainly in the perinuclear region in contrast to the control group, and the RhoA fluorescence-associated is equally widespread in the cytosol (Figure 5H–J).

**Figure 6:**
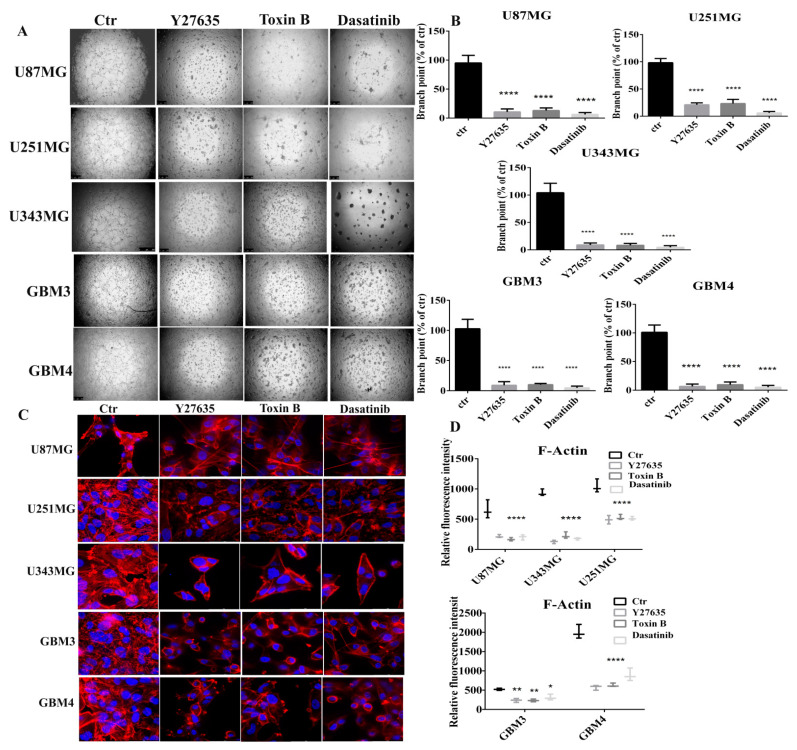
Effect of iPA on actin stress fibers. Inhibition of vasculogenic mimicry by Y27635 and Toxin B of (**A**) GBM stabilized and primary cell lines. Y27635, Toxin B, and Dasatinib inhibit vasculogenic mimicry in GBM and primary cells. (**B**) Branch point (sites of intersection of at least three tubes) number in each well was counted. Immunofluorescence staining of Actin (red signal, Phalloidin) to show changes in the levels of stress fibers in (**C**) GBM stabilized and primary cell lines. (**D**) The rate of F-actin was quantified and data are plotted as boxplots. Results are expressed as the mean ± SD of four independent experiments conducted in triplicate. (ANOVA, * *p* < 0.05, ** *p* < 0.01, **** *p* < 0.0001).

As the tube formation in VM is dependent on actin remodeling regulated by RhoA activity, we performed a tube formation assay in GBM lines and primary cells treated with Y27632, Toxin B, and Dasatinib. Y27632 is a selective inhibitor of ROCKI and II, an immediate downstream effector of RhoA [26], Toxin B, of *Clostridium difficile*, glycosylates threonine 37 residue of RhoA and threonine 35 residue of Cdc42 and Rac causing the inactivation of small GTPases [27]. As depicted in Figure 6A,B, Y23762 at 20 µM, Toxin B at 100 ng/mL, and Dasatinib at 1 µM treatment or 18 h significantly inhibited VM tube formation of GBM cell lines and primary cells. In addition, to confirm the role of RhoA activity and Src kinase in the actin organization we observed that treatment with Y23762, Toxin B, and Dasatinib reduced the actin stress fiber, as shown in Figure 6C. The rate of F-actin was quantified and the data are shown in Figure 6D.

## 3. Discussion

Glioblastoma is an aggressive malignancy highly vascularized and resistant to standard therapy. GBM progression is supported by alternative vascularization mechanisms including co-option of existing host vessels [28], angioblast vasculogenesis [29], intussusceptive angiogenesis [30], and vasculogenic mimicry, a mechanism described and defined by Maniotis et al. for malignant melanoma [31]. VM is a fluid conduction network made up of tumor cells, embedded in the matrix, independent of endothelial cells. For glioma, the VM process is associated with tumor invasiveness, migration, and poor prognosis. VM formation mechanism involves tumor cells that can express both endothelial and tumor phenotypes.

Vasculogenic mimicry occurs in GBM as an alternative vascularization mechanism, providing a means whereby GBM can escape anti-angiogenic therapies. From our previous studies, it has been seen that iPA can interfere in the angiogenic process through the activation of the AMPK pathway [16].We observed that iPA inhibits the vasculogenic mimicry in glioblastoma cells by disassembling of adherens junctions (AJ) and by reduction in the actin stress fibers. VE-cadherin, as well as E-cadherin are the key factors regulating cell–cell adhesion through AJ by anchoring and organizing the cortical actin cytoskeleton in the endothelium and epithelium. Moreover, they play an important role in the acquisition of VM capabilities of tumor cells [32]. Here, we demonstrated that iPA, an adenosine modified with isoprenoid derivatives, inhibited the VM formation of GBM cells lines, cancer stem cells, and cell models derived by tumor biopsies of patients affected. iPA reduced the protein levels of VE-cadherin and E-cadherin by hindering the VM mechanism and inducing the AJ disassembly. Cadherins-mediated adherens junctions are key players in intercellular interactions. They improve cell coordination during collective migration; moreover, adherens junctions transduce signals participating to the control of directed cell migration and in the regulation of cellular functions, such as proliferation and differentiation [33]. Cadherins’ functions are controlled by catenins that interact with their cytosolic tail. We evaluated the iPA effect on two members of the catenins family: p120- and β-catenins. p120-catenin is important for the stability of E-cadherin at the plasma membrane and β-catenin is necessary for recruiting the actin cytoskeleton [34]. We observed that iPA inhibited the phosphorylation at Y228 residue of the p120-catenin, an Src kinase-site phosphorylation. In this study, we found Src kinase phosphorylation inhibition at Y416 residue by iPA treatment at 10 µM after 18 h. Src modulates adhesion site function affecting the phosphorylation status of AJ proteins and is highly activated in cancer regulating a wide variety of both normal and oncogenic processes including proliferation, adhesion, invasion, and motility [35]. To assess if the Src inhibition is responsible for VM inhibition, we used an Src inhibitor, Dasatinib, which, like iPA, is able to inhibit p120 catenin phosphorylation, the VM, and to reduce the actin stress fibers. Furthermore, p120-catenin Y228 phosphorylation has been associated with the progression of oral squamous cancer [35] and with the increased invasiveness of glioblastomas [36]. Results of this study suggest that iPA is able to decrease the invasive capability of glioblastoma cells. iPA treatment inhibited the β-catenin at S33/S37/T41 residues suggesting its release from AJ and nuclear accumulation [37]. β-catenin phosphorylation is regulated by GSK-3α/β which is inhibited at T219/216 residues by iPA treatment. Several results indicate that p120-catenin can also control the activity of small GTPases. Rho family GTPases such as Rho, Rac, and Cdc42 play important roles in the regulation of cytoskeletal organization and dynamics during cell spreading and migration [38]. Once activated, RhoA triggers a complex set of signal transduction pathways that include both the Rho-associated coiled-coil-containing protein kinase (ROCK) activation pathway, which is responsible for actin cytoskeleton remodeling, which affects cell motility, thereby supporting migration, invasion, and tube formation cellular processes associated with VM. In this study, we show that iPA affects the distribution and activation state of RhoA-GTPase and consequently actin organization. iPA at 10 µM caused an inhibition in the GTPase activity of RhoA. Therefore, treatment of GBM cells lines with iPA attenuated the translocation from the cytosol (inactive form) to the plasma membrane (active form) after 18 h. This event could provoke a significant reduction in F-actin formation determining the loss of traction forces required for cell motility. Indeed, we showed a reduction in the stress fibers after iPA treatment. We observed that the RhoA distribution changed: in untreated GBM cells lines, RhoA-associated fluorescence was located in the proximity of the plasma membrane, while in treated cells, RhoA-associated fluorescence was located in the perinuclear region and widespread in the cytosol, suggesting that iPA induced a delocalization of RhoA from the cell membrane to the cytoplasm and this effect determined a disruption of skeleton actin stress fibers. To gain further insight into the role of RhoA in the F-actin cytoskeleton remodeling, we examined the effect of Y23762, ROCK1 inhibitor, and Toxin B from *Clostridium difficile*, that inactivates the Rho family members (Rac, Cdc42, and RhoA) by glycosylation of threonine residues. We demonstrated that both the inhibition of ROCK1, downstream effector of RhoA signaling and the inactivation of GTPases inhibited the tube formation in GBM cells. Taken together, the data shown in this study suggest that RhoA/ROCK signaling could be involved in the maintenance of actin organization, induction of migration, and tube formation involved in the VM. We have also observed that Dasatinib induced RhoA activation, as recently described in endothelial cells [39], suggesting that the reduction in actin stress fiber could be due to Src inhibition. Moreover, in this study, we observed that Dasatinib, like iPA, is able to inhibit the p120 catenin/RhoA interaction allowing the disassembly of AJ. We guess that the inhibition of RhoA activity may also be due to the inhibition of the prenylation of the protein, as has been observed in our previous manuscript. iPA inhibits farnesyl diphosphate synthase activity causing in the cell a reduction in isoprenoid compounds, such as farnesylpyrophosphate (FPP) and its downstream product geranylgeranylpyrophosphate (GGPP), both essential for the activity of the prenylated proteins, including RhoA [40]. These findings show the pivotal role of Rho A in the cytoskeleton actin remodeling and cell migration. iPA, through inhibition of RhoA activity and its downstream effectors ROCKs, causes the disassembling of actin stress fibers suppressing cell migration and hindering the VM of the GBM cells. To support this hypothesis, we used lysophosphatidic acid (LPA), Rho activator [41]. As shown (Figure S5), lysophosphatidic acid (LPA) induced the VM in GBM cells and co-treatment with iPA (at concentration ranging 1 to 10 µM) and LPA at 10 µM of GBM cells inhibited the VM tube formation, suggesting the iPA inhibitory effect on Rho A activity.

Lastly, our data show that iPA inhibits Tyr416 phosphorylation of Src in GBM cells and subsequent Src kinase activity. Activated c-Src is important for cadherin function through a signaling cascade that controls processes such as cadherin accumulation and cytoskeletal reorganization mediated by the interactions with catenins thus supporting the adherens junction complex. The c-Src kinase pathway inhibition by iPA causes the disassembly of AJ through the phosphorylation inhibition of p120-catenin and reduction in protein levels of E-cadherin [29]. Moreover, iPA through inhibition of RhoA activity reduces the actin stress fiber and migration cells hindering VM formation. iPA may effectively block the blood supply to tumors, providing a rationale for more favorable targeted therapies in GBM treatment.

## 4. Materials and Methods

### 4.1. Drugs and Reagents

For all experiments, the solutions were prepared starting from the stock solution. N6-isopentenyladenosine (iPA), Y27632 was purchased from Sigma-Aldrich (St. Louis, MO, USA), solubilized in dimethyl sulfoxide (DMSO) (10 mM), and added to cell culture growth medium at different concentrations. Toxin B, *Clostridium difficile*, was purchased from Calbiochem, Sigma-Aldrich (St. Louis, MO, USA). Dasatinib was donated by Prof. Roberto Bianco [24]; lysophosphatidic acid (LPA) (CAS 325465-93-8) was purchased from Santa Cruz Biotechnologies (Santa Cruz, CA, USA).

### 4.2. Cell Cultures

Human glioblastoma stabilized cell lines, U87MG and U343MG, were purchased from Elabscience (catalog No. EP-CL-0238); U251MG cells were purchased from Elabscience (catalog No. EP-CL-0237). HUVEC was provided by Gibco Angiogenesis Starter Kit (Woltmann Massachusetts, USA). Cells were cultured in DMEM (Grand Island, NE, USA) supplemented with 10% heat-inactivated fetal bovine serum, 1% L-glutamine, 1% sodium pyruvate, 1% non-essential amino acid (Lonza, Rome, Italy), and 0.1% plasmocin TM prophylactic (InvivoGen, San Diego, CA, USA), at 37 °C with 5% CO2 atmosphere. For primary cell lines, the patients underwent tumor resection at the Neurosurgery Service of “Antonio Cardarelli” Medical Hospital (Napoli, Italy). All tissue samples were collected in accordance with the ethical standards of the Institutional Committee (DEL. N°897, 13 August 2020). Informed written consent was obtained from all subjects involved in the study. The samples of brain tissue containing tumor were immediately processed to obtain primary tumor cell lines (designated as GBM3 and GBM4) using gentleMACS™ Dissociator (Miltenyi Biotec, Cologne, Germany) in combination with the Tumor Dissociation Kit, human (Miltenyi Biotec, Cologne, Germany), following the manufacturer’s instructions. Two tumor samples (designated as GBM1 and GBM2) were obtained from Neurosurgery Dept. of IRCCS Ospedale Policlinico San Martino (Genova, Italy), after the approval of the Institutional Ethical Committee of the informed consent given from patients and of the ex vivo human sample study (ethic code 17/12, approved 14 September 2012) [13].

### 4.3. Primary Glioblastoma Characterization

For each tumor sample, the Illumina EPIC ARRAY 850 Bead-Chip (850 K) platform was used to evaluate the DNA methylation status of 850,000 CpG sites, following the manufacturer’s instructions. A reference cohort formerly analyzed at the German Cancer Research Center was compared to the epigenomic profile obtained from the samples, using a specific algorithm and customized bioinformatics packages as described previously. In addition, the array data were used to calculate a copy number variation profile, as previously described. Here, samples copy number profiles were used to verify the presence/absence of the EGFR gene. “Gain” or “amplification” was determined by log2 > 0.3.

### 4.4. IDH1 and IDH2 Mutation Status

Tumor DNA extracted from FFPE (Formalin-fixed, paraffin-embedded) tumor tissue (FFPE Tissue Kit, Qiagen, Germany) was amplified using specific primers for exon 4 of IDH1 and IDH2 genes. (IDH1: forward primer 5′-TGTAAAACGACGGCCAGTGGATGCTGCAGAAGCTATAA-3′; reverse primer 5′-CAGGAAACAGCTATGACCTTCATACCTTGCTTAATGGG-3′. IDH2: forward primer 5′-TGTAAAACGACGGCCAGTAATTTTAGGACCCCCGTCTG-3′; reverse primer 5′-CAGGAAACAGCTATGACCGGGGTGAAGACCATTTTGAA-3′). Codon 140 and 172 of IDH2 and codon 100 and 132 of IDH1 were then analyzed using the Sanger sequencing method.

### 4.5. MGMT Methylation Assessment

MGMT promoter methylation was determined by Methylation-specific PCR (MSP). DNA extracted from Tumor samples was converted by sodium bisulfite with EZ DNA Methylation Gold Kit (Zymo Research, Germany) according to the manufacturer’s protocol. Methylation-specific PCR was performed using a Nested PCR. The first PCR step was performed using specific primers 5′-GGATATGTTGGGATATAGTT-3′, reverse: 5′-CCATCCACAATCACTACAA). In the second PCR step, we used different primers for methylated and non-methylated DNA samples (methylated MGMT: forward primer 5′-TTTCGACGTTCGTAGGTTTTCGC-3′; reverse primer 5′- GCACTCTTCCGAAAACGAAACG-3’. Unmethylated MGMT: forward primer 5′-TTTGTGTTTTGATGTTTGTAGGTTTTTGT-3′; reverse primer 5′-AACTCCACACTCTT CCAAAAACAAAACA-3′) as previously described. As a positive control, we used a commercial methylated DNA for methylated MGMT alleles, and as a negative control, we used a non-methylated commercial control (EpiTEC controls from Qiagen). Controls without DNA were used for each set of MSP assay. Then, each MSP product was loaded directly onto 3% percent agarose gel, stained with ethidium bromide (Sigma-Aldrich, St. Louis, MO, USA), and examined under ultraviolet illumination using a ChemiDoc MP image sensor. (Biorad, Hercules, CA, USA).

### 4.6. Reagents and Antibodies

The antibodies used in the Western blot analysis were: mouse monoclonal anti-human β-actin, mouse monoclonal anti-human α-tubulin, mouse monoclonal anti-human E-Cadherin, mouse monoclonal anti-human p-GSK (T279/T216), mouse monoclonal anti-human p120 Catenin, and mouse monoclonal anti-human GLUT3 were purchased from Santa Cruz Biotechnology (Santa Cruz, CA, USA), rabbit monoclonal anti-human p-Src (pY416), rabbit monoclonal anti-human β-Catenin, rabbit monoclonal anti-human p-β-Catenin (S33/37/T41), rabbit monoclonal anti-human RhoA, and rabbit monoclonal anti-human p-AKT were purchased from Cell Signaling Technology (Danvers, MA, USA); rabbit monoclonal anti-human p-p120 catenin (pY228) from BD Biosciences (Franklin Lakes, NJ, USA). For fluorescence microscopy, the following were used as primary antibodies: rabbit monoclonal anti-human RhoA (Cell Signaling Technology); as secondary antibody Alexa Fluor^®^ 488 goat polyclonal anti-rabbit IgG (Jackson ImmunoResearch, Cambridge, UK) and DyLight^®^ 594 goat polyclonal anti-mouse IgG (Abcam, Cambridge, UK).

### 4.7. MTT Vitality Assay

The MTT colorimetric assay was used to assess cell viability. Cells at a density of 3 × 10^4^ were plated in p60 dishes. After treatment with iPA dissolved in culture medium (DMEM), the cells were incubated in a new medium in which 5 mg/mL of MTT (Sigma-Aldrich) was dissolved for sufficient time for the formation of purple crystals (about 30 min). Subsequently, the medium was removed, and acid-isopropanol (10% HCl 1N in isopropanol) was added to each plate to solubilize the crystals. After 20 min at room temperature in agitation, the samples were recovered, and cell mortality was assessed with a Synergy HT Microplate Reader (BioTek Instruments Inc., Winooski, VT, USA) HTX microplate reader using a wavelength of 570 nm.

### 4.8. Western Blot Analysis

The total protein was extracted using RIPA (Radioimmunoprecipitation assay) lysis buffer (50 mM Tris-HCl, 150 mM NaCl, 0.5% Triton X-100, 0.5% deoxycholic acid, 10 mg/mL leupeptin, 2 mM phenylmethylsulfonyl fluoride and 10 mg/mL aprotinin, supplemented with phosphatase and protease inhibitors (Sigma-Aldrich, St. Louis, MO, USA). Equal amounts of lysate were separated on SDS-PAGE gel and transferred onto nitrocellulose membranes using a Trans-Blot^®^ Turbo™ Transfer System (BioRad, Hercules, CA, USA). Upon saturation, primary antibodies were used to probe the proteins of interest overnight at 4 °C; the membranes were subsequently incubated with horseradish peroxidase (HRP)-conjugated secondary antibodies at room temperature for 1 h. Protein bands were detected using the ChemiDoc MP image sensor (BioRad, Hercules, CA, USA).

### 4.9. Cell Migration and Invasion Assay

Migration and invasion assays were performed using a Transwell assay. For the Migration assay, cells were seeded into the Transwell chamber (8 μm pore size; Costar, Cambridge, MA, USA) inserted in a 24-well plate, at a density of 5 × 10^4^ cells per well and cultured with DMEM without FBS, while DMEM containing 10% FBS was added in the lower chamber. After 24 h, cells were fixed with 4% paraformaldehyde, stained with 0.5% crystal violet (12 mM in MeOH 20%), and each well was photographed using a Leica DMi8 M System fluorescence microscope. The migrating cells were quantified by counting the stained cells using ImageJ.Ink software (1.51i, NIH, Bethesda, MD, USA). The cell invasion assay was performed similarly to the migration assay with the only difference that the filter membrane was first coated with Geltrex™ (Gibco, Thermo Fisher Scientific, Waltham, MA, USA), while the number of cells seeded was 2.5 × 10^4^ per well.

### 4.10. In Vitro Tube Formation Assay

Angiogenesis was measured using a tube formation assay. Cells were seeded in 96-well plates, pre-coated using 50 µL/well of Geltrex™ (Gibco) with ECM proteins concentration of 10 mg/mL. The coating was allowed to polymerize at 37 °C for 30 min. The cells were dissociated by trypsinization, washed in PBS (+Mg^2+^, +Ca^2+^), and re-suspended in serum-free medium at 3 × 10^4^ cells/mL. Subsequently, 0.1 mL/well cell suspensions were plated onto the surface of Geltrex™ and incubated at 37 °C for 24 h. Following incubation, each well was analyzed directly under a Leica DMi8 M System fluorescence microscope, tubes in each field were imaged and the number of branch sites/nodes (sites of intersection of least three tubes) was counted using the ImageJ.Ink software (1.51i, NIH, Bethesda, MD, USA). The average branch point number from 5–6 random fields in each well was reported.

### 4.11. Subcellular Fractionation

Subcellular fractionation was performed following the protocol by Bio-Protocol (https://bio-protocol.org/e754, accessed on19 April 2021). Culture cells were washed with ice-cold PBS and SF (Subcellular fractionation) buffer was added. Using a cell scraper, the lysate was collected and transferred to a 1.5 mL Eppendorf tube. After various centrifugations, following the manufacturer’s instructions, the membrane and cytosolic fractions were obtained, and the samples were resolved on SDS-PAGE gel as stated before.

### 4.12. Immunofluorescence Staining

Cells were seeded and cultured in 24-well culture plates at a density of 1 × 10^5^ cells per cm^2^ and treated with iPA. For immunofluorescent staining, cells were fixed with 3.7% paraformaldehyde, permeabilized with 0.2% Triton X-100 and blocked using PBS-BSA 0.4%. Subsequently, cells were incubated with primary antibody at 4 °C overnight. The next day, cells were incubated with a labeled secondary antibody at room temperature for 1 h. DAPI (Hoechst, Life Technologies Corporation, Thermo Fisher Scientific, Waltham, MA, USA) in PBS was used for nuclei staining and rhodamine-phalloidin was used as the actin staining reagent (purchased from Abcam, Cambridge, UK). Finally, cells were mounted on slides using Dako Fluorescent Mounting Medium. The images were acquired using a Leica DMi8 M System fluorescence microscope.

### 4.13. Active Rho Pulldown Assay

The evaluation of active Rho was performed using the Active Rho Detection Kit (Cell Signaling Technology, Danvers, MA, USA), according to the manufacturer’s protocol. At least 500 µg of total protein was used to pull down active Rho with 400 µg of GST-Rhotekin-RBD. The active protein signal was detected through Western Blot Analysis, as described earlier.

### 4.14. Immunoprecipitation

For immunoprecipitation assays, the growth medium was removed, the cells were washed in cold phosphate-buffered saline (PBS) and placed in RIPA lysis buffer as explained previously. Specific amounts of protein were immunoprecipitated using protein A-agarose beads with a specific primary antibody overnight at 4 °C. The samples were then washed, added to 25 μL of Laemmli (BioRad, Hercules, CA, USA), and the supernatants were used for Western Blot analysis as explained earlier.

### 4.15. Statistical Analysis

Statistical analysis was performed using GraphPad Prism 7.0 software for Windows (GraphPad software San Diego, CA, USA). The data were reported as the mean ± SD and analyzed for statistical significance using the two-tailed Student *t*-test, for independent groups, or ANOVA followed by Bonferroni correction for multiple comparisons. A *p*-value < 0.05 was considered statistically significant.

## 5. Conclusions

**Figure 7:**
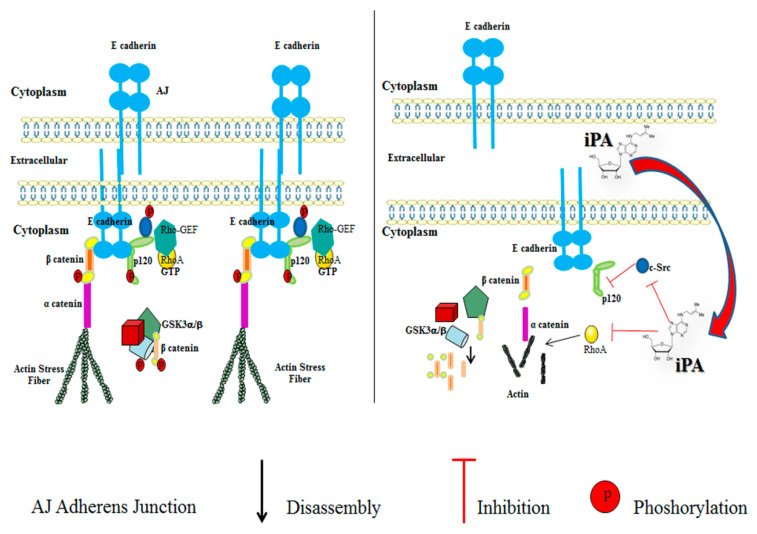
Schematic description of cells before and after iPA treatment. The major component of the AJs are the E-cadherins, which can pair with the E-cadherins exposed on the opposing cell. The mechanism leading up to adherens junction complex are still not clear, but they derive from the recruitment of cadherins, catenins (beta-catenins, alpha-catenins), and cytoskeletal proteins. Catenins and p120 catenin in their phosphorylated forms bind to the cytoplasmatic tail of the cadherins. A hypothetical model proposes that p120, phosphorylated by c-Src, can indirectly regulate RhoA activity. At the same time, β-catenin, through α-catenin, recruits the actin cytoskeleton, strengthening the cell compaction with the formation of stress fibers [42] iPA treatment causes the inhibition and reduction in proteins involved in the AJs assembly. We showed that iPA inhibits c-Src kinase activity through the decrease in c-Src phosphorylation. This event leads to the inactivation of p120 catenin and the inhibition of GSK3 α/β and accordingly of α-catenin. In addition, iPA reduced the levels of E-cadherin determining the AJ disassembly. iPA, through the inhibition of RhoA activity, induces the reduction in actin stress fibers inhibiting cell migration, invasion, and VM formation.

The current study indicates that iPA inhibits migration, invasion, and VM formation of glioma through a mechanism likely related to the suppression of the c-Src pathway and Rho signaling. These results demonstrate that iPA disarranges the adherens junctions by interfering with the Src-120 catenin pathway, thereby preventing the tube formation in the VM mechanism. In contrast, the Rho/ROCK signaling inhibition provokes the F-actin disorganization contributing to reduced migration and invasion of the glioma cells. iPA acts as a multi-target inhibitor thus representing a promising therapeutic tool for the treatment of glioblastoma (Figure 7).

## Figures and Tables

**Table 1:** Reassuming the genetic and epigenetic profile of GBM3 and GBM4 primary cell lines obtained from affected patients.

GBM	Patient		Type	MGMT Methylation Status		IDH1/IDH2 Status		Co-del 1p-19q	Molecular Characterization	EGFR Amplification	Epigenetic Sub-Class
	Age	Gender		Patient	GBM Line	Patient	GBM Line				
GBM3	54	M	Primary	Unmethylated	Unmethylated	IDH WT	IDH WT	absent	GBM IDH WT	yes	RTKII
GBM4	64	M	Primary	Unmethylated	Methylated	IDH WT	IDH WT	absent	GBM IDH WT	no	Mesenchymal

## Data Availability

The data presented in this study are available on request from the corresponding author.

## Supplementary Materials

The following are available online at https://www.mdpi.com/article/10.3390/ijms221910530/s1.

## Abbreviations

3D	Three-dimensional
AJ	Adherens Junction
AMPK	5’ adenosine monophosphate-activated protein kinase
ANOVA	Analysis of variance
BSA	Bovine serum albumin
Cdc42	Cell division control protein 42
CSCs	Cancer stem cells
c-Src	Proto-oncogene tyrosine-protein kinase Src
DAPI	6-diamidino-2-phenylindole
DMEM	Dulbecco’s Modified Eagle’s Medium
DMSO	Dimethyl sulfoxide
E-Cadherin	Epithelial cadherin
ECM	Extracellular Matrix
EGFR	Epidermal growth factor receptor
FBS	Fetal bovine serum
FFPE	Formalin-fixed, paraffin-embedded
FITC	Fluorescein isothiocyanate
FPP	Farnesylpyrophosphate
GBM CSC	Glioma stem cell lines
GBM	Glioblastoma
GGPP	Geranylgeranylpyrophosphate
GSK	Glycogen synthase kinase
GST	Glutathione S-transferase
HCC	Human hepatocellular carcinoma
HRP	Horseradish peroxidase
HUVEC	Human umbilical vein endothelial cells
IDH1/2	Isocitrate dehydrogenase (NADP(+)) 1⁄2
iPA	N6-isopentenyladenosine
MGMT	O6-methylguanine DNA methyltransferase
MSP	Methylation-specific PCR
MTT	3-(4,5-dimethylthiazol-2yl)-2,5-diphenylterazolium bromide
PBS	Phosphate-buffered saline
PCR	Polymerase chain reaction
RhoA	Ras homolog family member A
Rhotekin-RBD	Rho binding domain (RBD) of the human Rhotekin
RIPA Buffer	Radioimmunoprecipitation assay buffer
ROCK	Rho-associated protein kinase
SD	Standard deviation
SDS-PAGE	Sodium dodecyl sulphate–polyacrylamide gel electrophoresis
SF buffer	Subcellular fractionation buffer
TRITC	Tetramethylrhodamine
VE-Cadherin	Vascular endothelial cadherin
VM	Vasculogenic mimicry
