# Supplementary material for: N6-Isopentenyladenosine Hinders the Vasculogenic Mimicry in Human Glioblastoma Cells through Src-120 Catenin Pathway Modulation and RhoA Activity Inhibition
Source: Int J Mol Sci. 2021 Sep 29;22(19):10530. doi: 10.3390/ijms221910530 (PMC8508824; doi:10.3390/ijms221910530)
Supplement: Supplementary file 1 [file ijms-22-10530-s001.zip › ijms-1348021-supplementary.pdf]

Supplementary Material

N6-Isopentenyladenosine Hinders the Vasculogenic Mimicry in Human Glioblastoma Cells through Src-120 Catenin Pathway Modulation and RhoA Activity Inhibition

Cristina Pagano [1], Giovanna Navarra [1], Olga Pastorino [1], Giorgio Avilia [1], Laura Coppola [1], Rosa Della Monica [1,2], Lorenzo Chiariotti [1,2], Tullio Florio [3,4], Alessandro Corsaro [3], Giovanni Torelli [5,6], Pasquale Caiazzo [6], Patrizia Gazzerro [7], Maurizio Bifulco [1,*,†] and Chiara Laezza [8,*,†]

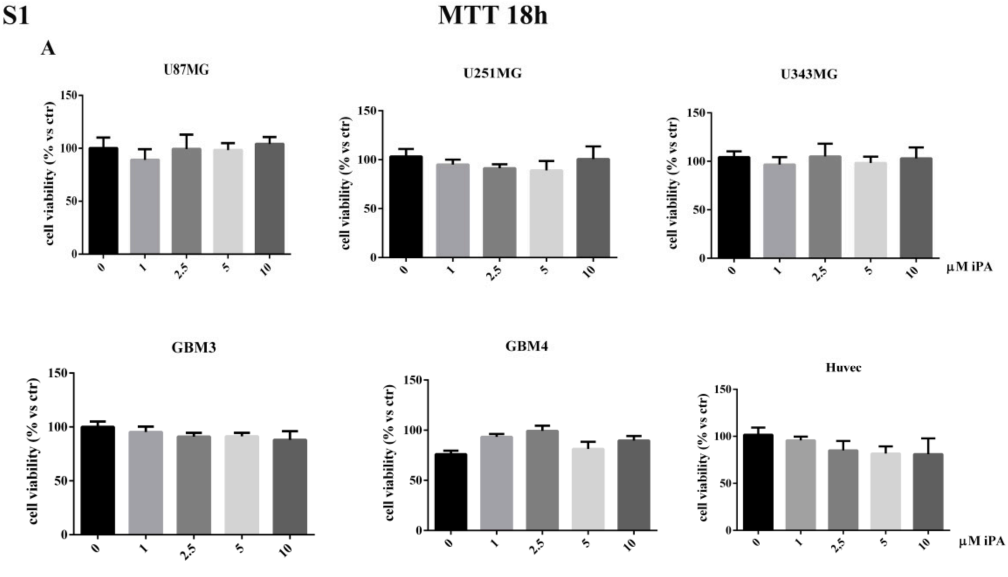

Supplementary Figure S1 (A) The cell rate viability assayed by MTT assay of U87 MG; U251 MG; U343 MG; GBM3; GBM4; Huvec cell lines. At several concentration of iPA no differences at 18 hr was detected in treated groups as compared to the control group. ANOVA*p < 0.05. **p < 0.01***p < 0.001 ****p < 0.0001.

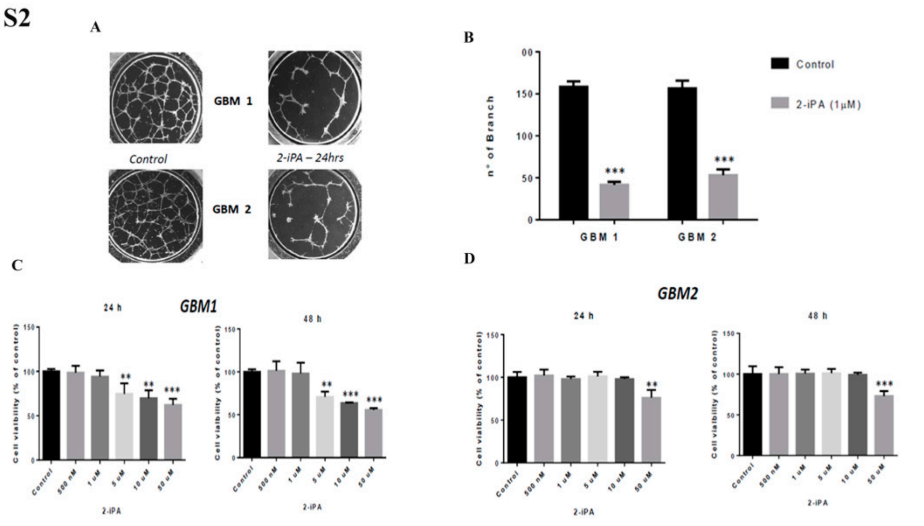

Supplementary Figure S2 (A) Tube formation of GBM1 and GBM2 (GBM CSCs). iPA treated cells showed a reduction in the tube formation process after 24h of treatment. (B) Histograms showing that iPA 1uM reduced the of number of branch points in GBM1 and GBM2. (C),(D) Graphic representation of MTT assays used to determine cell viability of GBM1 and GBM2 glioblastoma stem cell lines treated with different concentrations (500 nM-50μM) of iPA for 24 and 48 h of treatment. ANOVA *$p < 0.05$. **$p < 0.01$***$p < 0.001$ ****$p < 0.0001$.

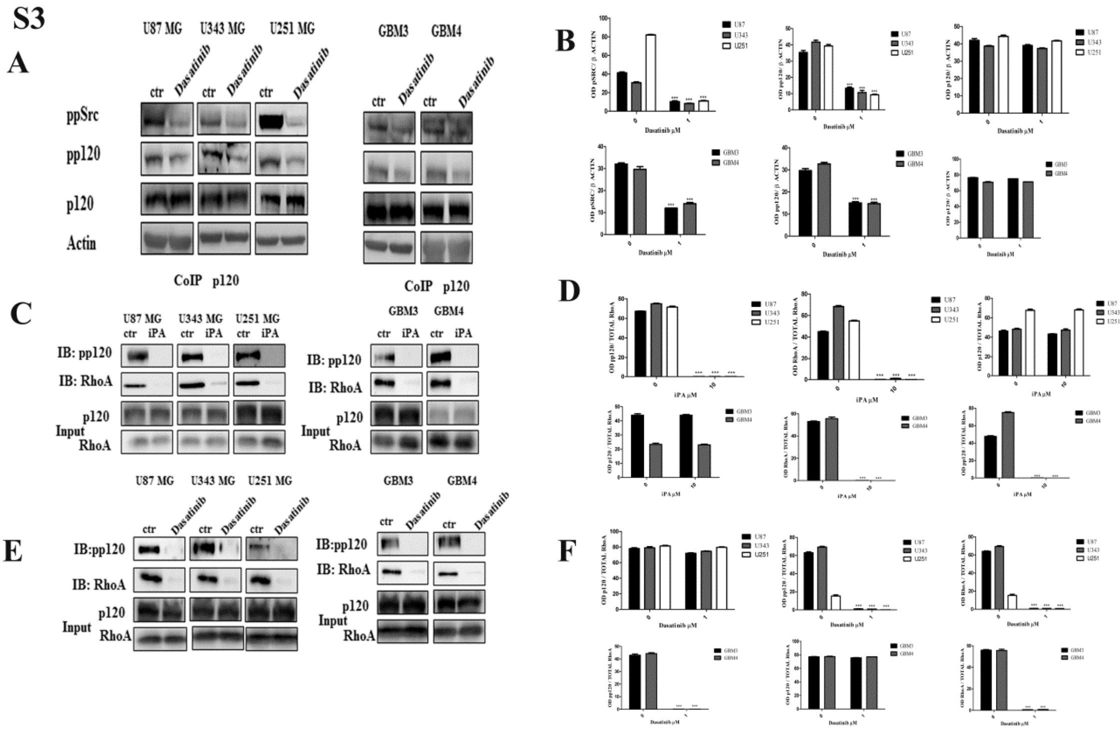

Supplemantary Figure S3 (A)The levels of ppSrc, pp120 and p120 were determined by Western Blot and β-actin was used as a loading control. As shown in the figure, Dasatinib 1 μM reduced the levels of both ppSrc and pp120 in all GBM cell lines used. (C)Western Blot showing the effects of iPA 10 μM on p120 and RhoA interaction after co-immunoprecipitation. The blots were revealed using anti-pp120 and anti-RhoA antibodies. As shown in the figure, pp120 do not coimmunoprecipitate with RhoA in all GBM cell lines treated with iPA 10 μM. (E) Western Blot showing the effects of Dasatinib 1 μM on p120 and RhoA interaction after co-immunoprecipitation. As before, the blots were revealed for pp120 and RhoA. Similarly to what happens after iPA treatment, pp120 do not coimmunoprecipitate with RhoA in all GBM cell lines treated with Dasatinib 1 μM. (B)(D)(F) Panels show the respective densitometry analysis of blots reported in (A)(C)(E). ANOVA *p < 0.05. **p < 0.01***p < 0.001 ****p < 0.0001

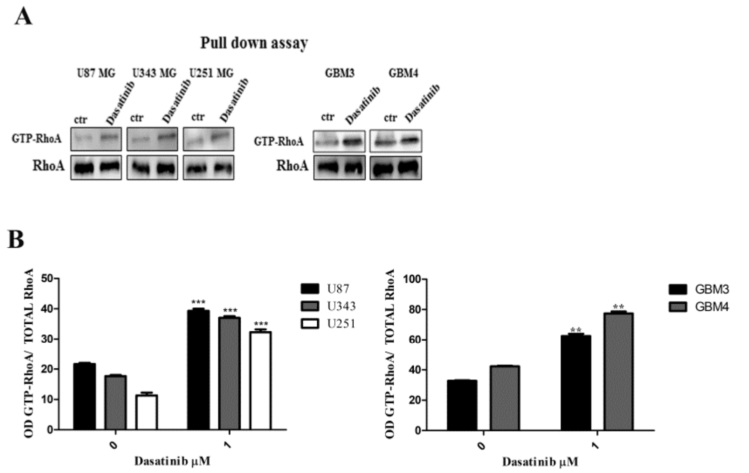

Supplementary Figure S4 (A)Western Blot showing the detection of GTP-RhoA by GST-Rhotekin-RBD pull-down assay in GBM cells treated with Dasatinib 1 μM. GTP-RhoA levels were increased after treatment with Dasatinib. (B) Densitometry analysis of blots reported in (A). ANOVA *p < 0.05. **p < 0.01***p < 0.001 ****p < 0.0001

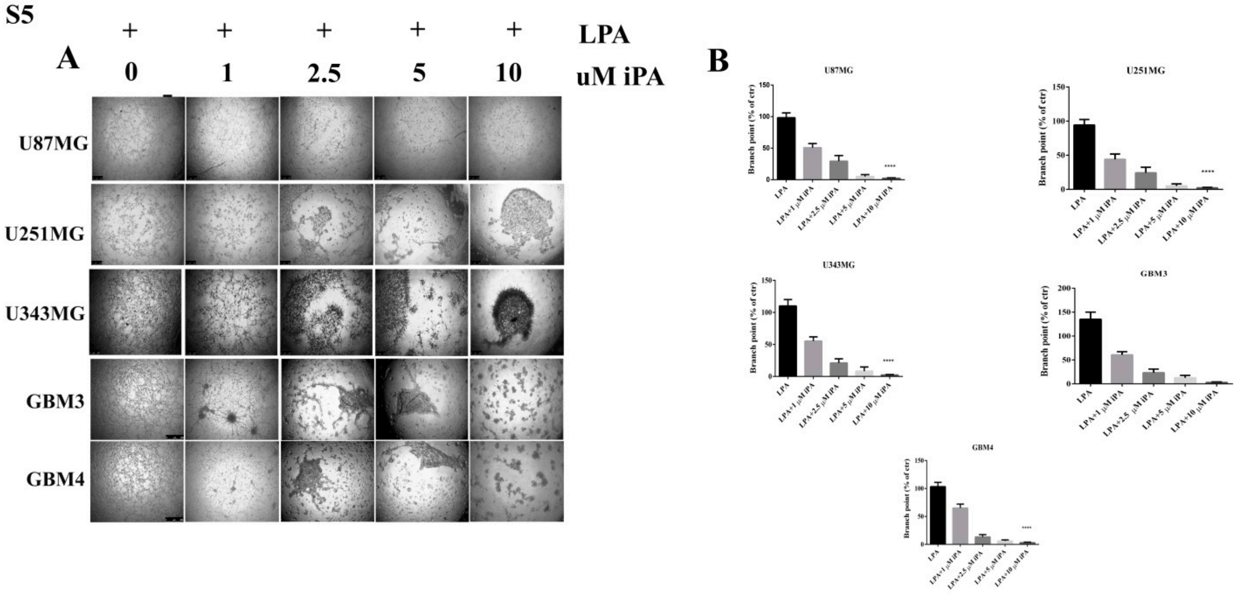

Supplementary Figure S5 : Effects of LPA and co-treatment with iPA on tube formation assay of GBM cells and primary cells line.(A) The tube formation assay shows the treatment of GBM cells, U87, U343, U251 and primary cell lines GBM3 and GBM4, with LPA 10 μM, for 1h, and after has been added iPA at different concentrations 1, 2.5, 5 and 10 μM for 18h.(B) The assay shows that the co-treatment with LPA and iPA inhibits the tube formations in GBM cells and primary cell lines, respect to only treatment with LPA.
